# Idiopathic Dilatation of Inferior Vena Cava: A Case Report

**DOI:** 10.7759/cureus.10313

**Published:** 2020-09-08

**Authors:** Ayesha Malik, Manahil Chaudhry, Mohammad Abdullah, Syed Hashim Ali Inam, Noreena Iqbal

**Affiliations:** 1 Internal Medicine, Combined Military Hospital Lahore Medical College and Institute of Dentistry, Lahore, PAK; 2 Internal Medicine, Hameed Latif Hospital, Lahore, PAK; 3 Internal Medicine, Army Medical College, Rawalpindi, PAK; 4 Internal Medicine, Milton Keynes University Trust Hospital, Milton Keynes, GBR

**Keywords:** idiopathic dilated ivc, dilatation of inferior vena cava, dilated ivc with celiac disease, dilated ivc in adult, dilated ivc with normal right atrial pressures

## Abstract

Inferior vena cava (IVC) is a large collapsible vein whose diameter and extent of inspiratory collapse are known to correlate with right atrial (RA) pressures; hence, IVC dilatation represents a cardiac pathology. IVC dilatation in the absence of any cardiac involvement is termed as idiopathic. We report a case of a 39-year-old male who presented with abdominal pain and had an incidental finding of isolated IVC dilatation with diminished inspiratory collapsibility and normal RA pressures. This case report emphasizes that IVC dilatation may not always have an underlying cardiac pathology.

## Introduction

Inferior vena cava (IVC) is a compressible vessel whose diameter is subjective to changes in both the intrathoracic pressure during breathing and the systemic venous return [1]. In most cases, the IVC diameter and its collapsibility reflect the right atrial (RA) pressures [2,3]. IVC diameter is lower during inspiration as compared to expiration [1]. The IVC diameter is affected by right heart function, as well as conditions like IVC aneurysm or Budd-Chiari syndrome (BCS), which directly or indirectly increase the volume of the blood in the right heart or increase the back pressure on the systemic circulation ultimately leading to IVC dilation [2,3].

Echocardiography is a non-invasive radiological investigation that can estimate various cardiac parameters and measure IVC diameter [3]. Under normal circumstances, the IVC diameter is less than 20 millimeters (mm) with a collapse of more than 50% during inspiration [4]. In cases of increased RA pressures, the collapsibility is diminished. Although detailed workup is warranted in cases of increased IVC diameter to find out the underlying etiology, increased IVC diameter is not always related to underlying pathology as there have been cases of young athletes who reported with dilated IVC diameters but with no underlying medical pathology. Our patient, known case of celiac disease, presented with abdominal pain and underwent an ultrasound abdomen, which revealed an incidental finding of dilated IVC with normal RA pressures on echocardiography done later. 

## Case presentation

A 39-year-old male, diagnosed case of celiac disease (Marsh 3b), presented to the emergency department (ED) with complaints of colicky abdominal pain and watery diarrhea for the past five days. The patient had no past history of any comorbidities, including diabetes mellitus, hypertension, ischemic heart disease, collagen vascular disease, congenital heart disease or dilated cardiomyopathy. Recent history was also not suggestive of any signs of infective diarrhea, and he claimed to have been very compliant with the dietary and lifestyle modifications for celiac disease. On examination, his abdomen was soft and non-tender, and the rest of the general and systemic examination was unremarkable. A noticeable mention here is that his cardiovascular system examination was also unremarkable, with no jugular venous distention, no murmurs or gallop rhythm and a regular heart rate.

**Figure 1:**
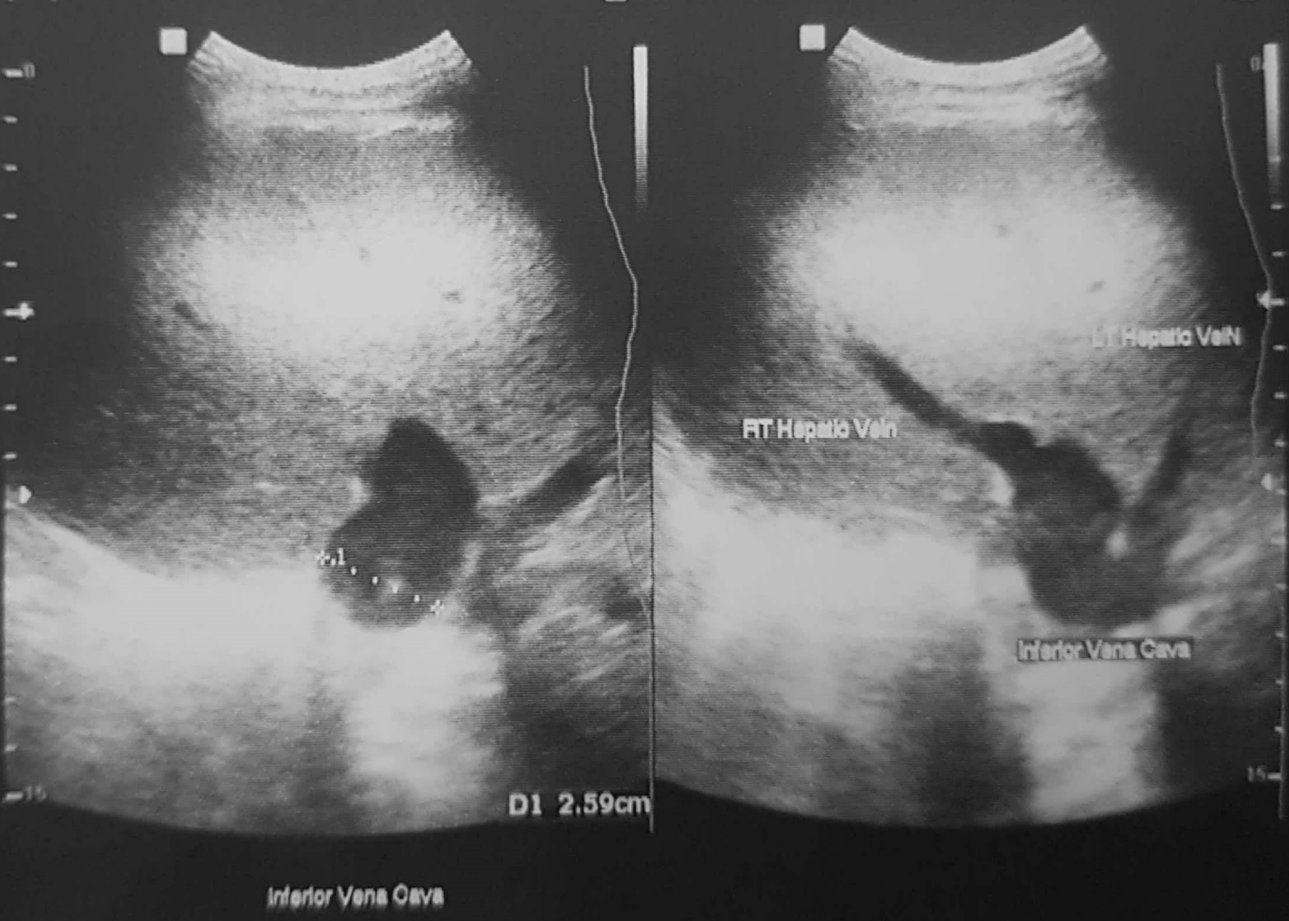
Inferior vena cava-hepatic vein confluence with caliper markings

Abdominal pain is a common symptom seen in celiac disease patients. However, due to the colicky nature of his pain, he was advised an ultrasound abdomen while in the ED. He was given intravenous antispasmodic injection and intravenous fluids. Additionally, his baseline labs, including complete blood count, liver function test, renal function test, urine routine examination, stool routine examination and microscopy (for ova and cysts) and coagulation profile, were sent from the ED, and all results were within normal limits. His ultrasound abdomen reported a normal study with no evidence of gallstones, pericholecystic fluid or inflammation of the pancreas. However, the study did show an incidental finding of dilated IVC (Figure 1).

The patient was advised an oral antispasmodic and oral hydration on discharge. Safety netting in case of worsening diarrhea was given. To further probe into the ultrasound finding, it was decided to pursue further investigations on an outdoor basis. On the follow-up visit, a transthoracic echocardiography was done and IVC was localized using the subxiphoid approach along the longitudinal axis. The scan was essentially normal except for a dilated IVC with no significant change in diameter, i.e. no collapse during inspiration and/or expansion during expiration. The study also revealed a normal pulmonary artery pressure (PAP), normal tricuspid annular plane systolic excursion (TAPSE), normal RA pressure (0-5 mmHg) and no evidence of tricuspid regurgitation (on flow Doppler). Right ventricle size and contractility were normal. No clot or thrombus was seen. Left ventricle ejection fraction was 60%. His abdominal pain and diarrhea had resolved on the follow-up visit.

**Figure 2:**
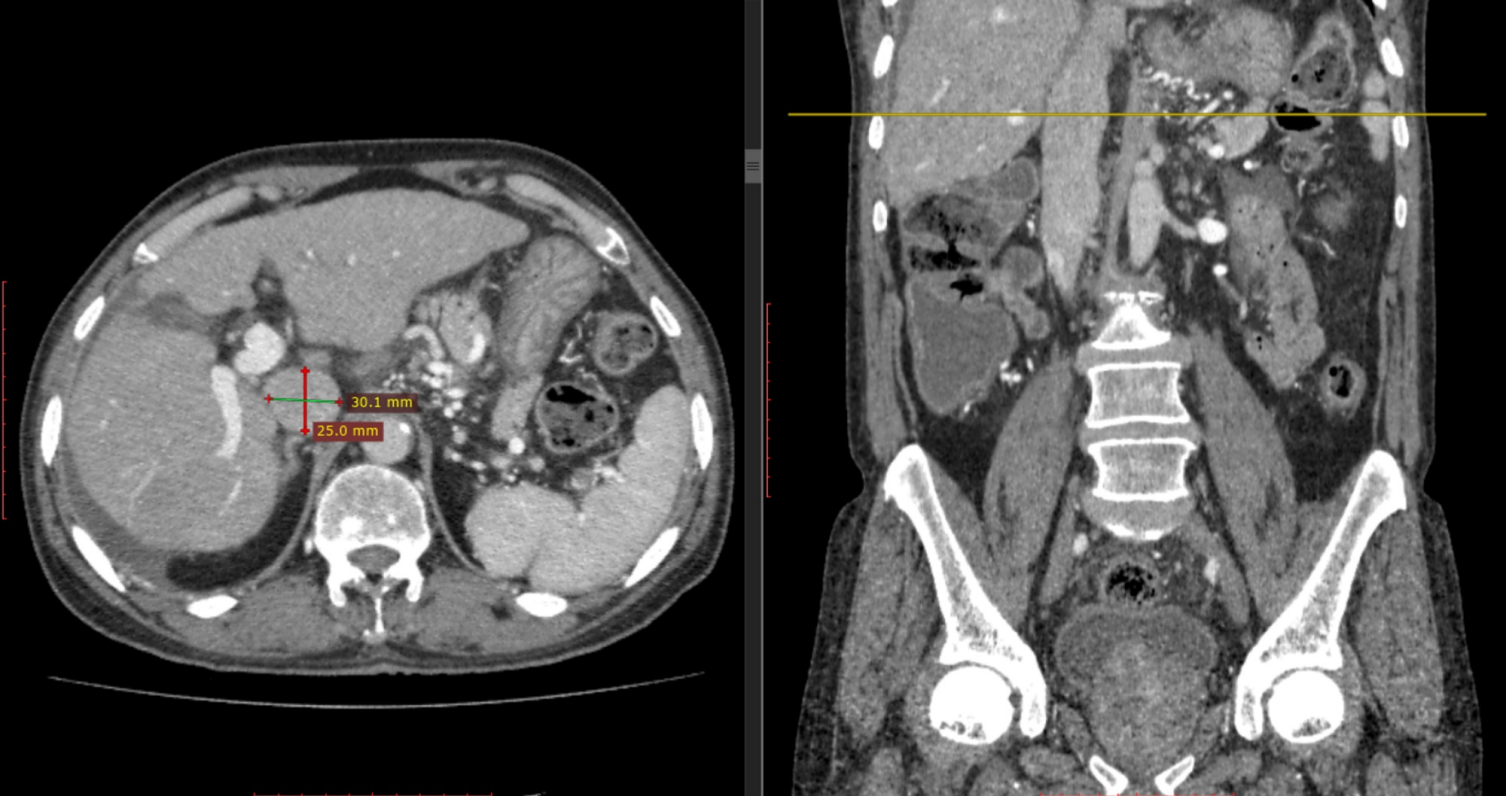
Transverse section showing dilated inferior vena cava

A duplex Doppler ultrasound for portal and hepatic vein flow was also done, which was a normal study and revealed no thrombosis. To augment the echocardiography results and rule out other possible causes of a dilated IVC, a contrast-enhanced triphasic CT (CECT) abdomen was pursued by the team, which reported a dilated IVC, dilated hepatic veins and internal iliac vein with IVC diameter being 30 mm x 25 mm at confluence and 20 mm x 12 mm caudally (Figure 2).

The scan showed no evidence of any abdominal mass and liver cirrhosis or BCS, and the portal vein diameter was also normal. After assuring no plausible causes of dilated IVC existed, the patient was given the diagnosis of an idiopathic IVC dilation. 

## Discussion

IVC diameter correlates with RA pressure [5]. Dilated IVC with no collapse is seen in acute heart failure and is associated with a higher six-month mortality. The cut-off diameter after which further investigation is warranted is 20 mm, and our patient had an IVC diameter of 30 x 25 mm (markedly increased) with no evidence of any right heart disease on echocardiography, including tricuspid regurgitation. Echocardiographic estimation of RA pressure is based on size and inspiratory collapse of IVC [6]. Maximum diameter 12 mm and collapse >50% suggest normal RA pressure (around 5-7 mmHg) [7]. Maximum diameter up to 20 mm and collapse >50% suggest RA pressure around 10 mmHg. Maximum diameter up to 20 mm and collapse 50% suggest RA pressure around 15 mmHg. Maximum diameter >20 mm without inspiratory collapse suggests RA pressure of >15 mmHg.

The CECT abdomen (triphasic flow) scan showed no evidence of IVC aneurysm. Neither ultrasound abdomen nor CECT abdomen showed evidence of any liver pathology like cirrhosis, space occupying lesion or BCS, which could contribute to a dilated IVC.

A study by Goldhammer et al. reported dilated IVC in athletes without heart disease [8]. Another case study by Gadi et al. also mentioned the association of IVC enlargement in highly trained athletes and patients with large body-surface area [4]. However, our patient was a middle-aged man with average build and with a rather sedentary lifestyle. Another feature of dilated IVC without collapse is its association with increased mortality in men and is usually seen in older males (70 ± 12 years) and is more frequently associated in patients with history of heart failure [9]. Conversely, our patient had neither of these traits. A rare finding seen in celiac patients is the presence of cardiomyopathy which may cause dilated IVC [10,11]. This too was not present in our patient as confirmed by echocardiography.

A recent study suggested that dilated IVC in healthy subjects might be a marker of decreased abdominal venous tone and/or increased compliance and correlated dilated IVC with incidence of vasovagal syncope [12]. When asked about any episode of light headedness or syncope on follow-up visits, the patient refused to ever have experienced so.

While a few cases have been reported worldwide with IVC dilation as an incidental finding, as per our knowledge this case is a first reporting of dilated IVC with celiac disease in Pakistan.

## Conclusions

Our patient had a dilated IVC with no underlying cardiac, congenital or any other identifiable pathology that could have led to the dilation. This case study concludes that dilated IVC may not always be due to some secondary pathology. However, further research studies on the prevalence and mechanism behind idiopathic dilated IVC cases can expand our recommendations and enable us to better understand this condition.
